# Drought-resistant trait of different crop genotypes determines assembly patterns of soil and phyllosphere microbial communities

**DOI:** 10.1128/spectrum.00068-23

**Published:** 2023-09-27

**Authors:** Baobei Guo, Hong Zhang, Yong Liu, Jianwen Chen, Junjian Li

**Affiliations:** 1 Institute of Loess Plateau, Shanxi University, Taiyuan, Shanxi, China; 2 Pomology Institute, Shanxi Agricultural University, Taiyuan, Shanxi, China; Pennsylvania State University, State College, Pennsylvania, USA

**Keywords:** water stress, crop microbiomes, community assembly, crop enzyme activity, co-occurrence networks

## Abstract

Crop microbiomes are widely recognized to play a role in crop stress resistance, but the ecological processes that shape crop microbiomes under water stress are unclear. Therefore, we investigated the bacterial communities of two oat (*Avena sativa*) and two wheat (*Triticum aestivum*) genotypes under different water stress conditions. Our results show that the microbial assemblage was determined by the crop compartment niche. Host selection pressure on the bacterial community increased progressively from soil to epiphyte to endophyte pathways, leading to a decrease in bacterial community diversity and network complexity. Source tracing shows that soil is the primary source of crop microbial communities and that bulk soil is the main potential source of crop microbiota. It filters gradually through the different compartment niches of the crop. We found that the phyla Actinobacteria, Proteobacteria, Gemmatimonadota, and Myxococcota were significantly enriched in bacterial communities associated with crop-resistance enzyme activity. Crop genotype influenced the composition of the rhizosphere soil microbial community, and the composition of the phylloplane microbial community was affected by water stress.

**IMPORTANCE:**

In this paper, we investigated the assembly of the plant microbiome in response to water stress. We found that the determinant of microbiome assembly under water stress was the host type and that microbial communities were progressively filtered and enriched as they moved from soil to epiphyte to endophyte communities, with the main potential source being bulk soil. We also screened for bacterial communities that were significantly associated with crop enzyme activity. Our research provides insights into the manipulation of microbes in response to crop resistance to water stress.

## INTRODUCTION

Drought has the greatest impact on agricultural productivity of all natural disasters because of its high correlation with crop growth and health and its expansive, prolonged disruptive effects (1, 2). The severity and duration of drought can lead to yield losses greater than those caused by all other factors combined (3). Improving crop resilience to future droughts by manipulating microbes that are closely related to the crop is a viable and sustainable approach (4, 5). The development of this effective technology is dependent on a fundamental understanding of the ecological processes involved in the microbiome assembly of the crop under water stress (6). However, few studies have investigated the adaptation of the crop bacterial microbiome to water stress.

Plant species and genotypes, soil type, environmental conditions, agricultural practices, and the interaction of these factors determine the assemblage of the plant microbiome (7 – 9). For example, the type of plant microbiome in a specified area can be divided into several categories based on the effects of plants and geographic conditions (10). The plant host strongly influences the microbiome assembly by providing a variety of environments, including epiphyte and endophyte tissues (11 – 13). The priority effect on colonization is also an important factor affecting the assembly of the plant microbiome as small differences in the early colonization process can result in large differences in community structure (14, 15). The soil biota plays an important role in the assembly of the plant microbiome as its main origin (16). For example, some studies conclude that soil factors rather than the land use type are responsible for the major differences in bacterial communities (17). Other studies have also shown that biogeography is the primary factor affecting microbial communities (18). In summary, the plant microbial community is influenced by diverse and complex processes, and identifying causal factors is challenging.

Soil moisture affects plant growth while also shaping the microbial communities associated with plants. Many studies have demonstrated that the functions, composition, and diversity of soil microbial communities are influenced when water stress events occur (19, 20). Generally, the diversity of the root microbial community was reduced (21, 22). The increased ratio of Gram-positive to Gram-negative bacteria was confirmed by different types of experiments (23). Analysis of the co-occurrence of bacterial community networks under drought conditions showed that the stability of the soil bacterial co-occurrence network was destabilized (24). Unfortunately, the factors that influence the establishment of plant-associated microbiomes are complex and interrelated, such that our understanding is rather limited. In particular, little is understood about how changes in microbial community assembly occur in the soil–plant continuum (25).

In this study, we analyzed the effects of water stress and host (compartment niche and host genotype) on the assemblies of crop microbiomes along the soil–plant continuum, including epiphytes (rhizosphere and phyllosphere) and endophytes (root and leaf), and identified keystone taxa and potential sources of the crop microbiome. We also investigated changes in the microbial assembly in different compartment niches with water stress; these compartments included phylloplane, leaf endosphere, rhizoplane, root endosphere, rhizosphere soil, and bulk soil. Finally, bacterial taxa significantly associated with crop enzyme activity were identified to corroborate the observed changes in bacterial communities under water stress. We hypothesized that the effects of the host and water stress on the crop microbial community assemblage would be transferred from the soil to the epiphytes and endophytes, resulting in reduced microbial diversity and network complexity. From the bulk soil to the endophytic part of the crop, the crop microbiome is gradually filtered and enriched, and the bacterial species in the endosphere were selected from the nearby compartments.

## MATERIALS AND METHODS

### Experimental design and treatments

We conducted a laboratory experiment investigating the mechanisms that determine the crop microbiome assembly under different levels of water stress. We selected two genotypes of oats (*Avena sativa*), Bayan-6 and Bayou-3, and two genotypes of wheat (*Triticum aestivum*), Jimai-22 and Jinmai-47, for our study. In general, oats exhibited a higher tolerance to water stress compared with wheat. Specifically, within the oat genotypes, Bayan-6 demonstrated a higher tolerance to water stress than Bayou-3. Similarly, within the wheat genotypes, Jinmai-22 exhibited a higher tolerance to water stress than Jinmai-47. The field soil was collected from the Dongyang Experimental Demonstration Base of the Shanxi Academy of Agricultural Science (37°33′16.5″N, 112°40′38.6″E, Shanxi province) and filtered through a 2-mM sieve. According to the FAO soil classification system (FAO-90) (26), the soils at this site are calcaric fluvisols with a pH of 8.3. The crops were planted in pots (depth: 8 cm; width: 7 cm) and raised under laboratory conditions (temperature: 24°C, natural light, and water every 2 days). There were 20 pots of each genotype making a total of 80 pots. These were divided into four water stress treatment groups, with five replicates for each treatment (Fig. S1). The water stress treatments began 3 weeks after planting. The soil’s water-filled pore space (WFPS) was calculated (27). The four water stress treatments were as follows: (i) crops flooded at all times; (ii) soil WFPS at 42% (control); (iii) soil WFPS at 30% (moderate drought stress); and (iv) soil WFPS at 20% (severe drought stress). The soil moisture content was measured twice daily using a ML3 Thetakit (Delta-T Devices, Cambridge, UK). The required soil moisture content for each treatment was maintained based on the observed WFPS values.

### Sample collection

Soil and crop samples were collected after 21 days of water stress treatment. Fresh crop leaves from five replicates of each treatment were collected to determine the superoxide dismutase (SOD), peroxidase (POD), and catalase (CAT) contents, and each measurement was repeated three times as technical replicates (*n* = 48). The collected leaf samples were immediately placed on ice (2 g, fresh weight [FW] for each plot). Samples of the crops’ rhizosphere soil (tightly attached to the roots) and roots (1–2 g FW for each plot) were then collected. Topsoil away from the roots was collected as bulk soil. Samples of leaves, roots, and soil were stored at −80°C for later DNA extraction. The available potassium and phosphorus, organic matter, pH, total N, $NH_{4}^{+}-N$ , and $NO_{3}^{-}-N$ in the soil were determined using established techniques (28), and these values are included in Table S1.

### Determination of enzyme activity in crop leaves

The activities of SOD, POD, and CAT in crop leaves were determined by the Activity Assay Kit, Micromethod (Sangon Biotech Co. Ltd.) using the multimode plate reader VICTOR Nivo (PerkinElmer, GER).

### DNA extraction and amplification of the 16S rRNA gene in bacteria

Samples for epiphytic DNA extraction were obtained from 2-g leaf and root samples. Microbial cells were dislodged and collected from the leaf surface and roots using methods as previously described (29), with some modifications, and subjected to DNA extraction using the E.Z.N.A. Soil DNA Kit (Omega Bio-Tek, Norcross, GA, USA) as directed by the manufacturer. Leaf and root endophytic DNA was extracted after further surface sterilization (29, 30). The DNeasy PowerSoil Pro Kit was used to extract rhizosphere and bulk soil DNA from 0.5 g of soil. An ABI GeneAmp 9700 PCR (ABI, CA, USA) thermocycler was used to amplify the hypervariable region V5-V7 of the bacterial 16S rRNA gene using primer pairs 799F (5′-AACMGGATTAGATACCCKG-3′) and 1193R (5′-ACGTCATCCCCACCTTCC-3′) (31). The 799F and 1193R primers do not amplify plant chloroplast DNA, and the resulting mitochondrial 800-bp product is easily eliminated, well excluding amplification of plant plastids (32). PCR amplification was performed in a total reaction volume of 30 µL containing 15 µL Phusion High-Fidelity PCR Master Mix, 0.2 µM forward and reverse primers, and 10 ng template DNA. Thermal cycling consisted of an initial denaturation at 98°C for 60 s, followed by 30 cycles of denaturation at 98°C for 10 s, annealing at 50°C for 30 s, extension at 72°C for 60 s, and finally 72°C for 6 min. The PCR product was purified and quantified using the AxyPrep DNA Gel Extraction Kit (Axygen Biosciences, Union City, CA, USA) and extracted from a 2% agarose gel (Promega, USA). Purified amplicons were pooled in equimolar amounts and paired-end sequenced on an Illumina MiSeq PE300 platform (Illumina, San Diego, USA) by Majorbio Bio-Pharm Technology Co. Ltd. (Shanghai, China). More details concerning the DNA extraction methods are available in Methods S1.

### Processing of sequencing data

The raw 16S rRNA gene sequencing reads were quality filtered with fastp version 0.20.0 after demultiplexing (33) and merged by FLASH version 1.2.7 (34) according to the following criteria: (i) the 300-bp reads were truncated at any site receiving an average quality score of <20 over a 50-bp sliding window, and truncated readings shorter than 50 bp or readings containing ambiguous characters were discarded; (ii) the maximum mismatch ratio for the overlap region was controlled at 0.2, with overlapping sequences greater than 10 bp in length being assembled only, and reads that could not be assembled were discarded; (iii) and based on barcodes and primers, samples were distinguished according to the sequence direction, and exact barcode matching was performed while primer matching was performed with a two-nucleotide mismatch.

We identified and removed chimeric sequences within operational taxonomic units (OTUs) based on a 97% similarity cutoff (35, 36) using UPARSE version 7.1. Using RDP Classifier version 2.2 (37), the taxonomy of each OTU representative sequence was analyzed using the 16S rRNA database (e.g., Silva v138) with a confidence threshold of 0.7.

### Statistical analysis

The significance of different factors on community dissimilarity was tested with permutational multivariate analysis of variance (PERMANOVA) or nested PERMANOVA using the “adonis” function of the “vegan” package (38) in R (https://CRAN.R-project.org/package=vegan) and based on the Bray–Curtis distance. Bray–Curtis distance matrices were used to quantify the bacterial beta-diversity, which were then coordinated with nonmetric multidimensional scaling (NMDS). The co-occurrence network analysis was performed using the R packages “psych” and “reshape2” using the Spearman correlation matrix and visualized in Gephi (https://gephi.org/) (39). Statistically significant (*P* < 0.05) and strong relationships (Spearman’s *r* > 0.6 or *r* < −0.6) were observed. To determine the complexity of a network, previous studies have identified nodes of high degree and proximity to the central value as the hub nodes of a co-occurrence network (40, 41). A linear mixed model (LMM) was used to find the primary factors influencing bacterial alpha-diversity using the R package “lme4” (42). The potential sources of the crop bacterial populations in each host niche were estimated using the fast expectation–maximization microbial source tracking (FEAST) methodology (43, 44). The correlation between enzyme activity and OTU was calculated using the “corr.test” function of the “psych” package in R. Bacterial functional profiles were predicted using functional annotation of prokaryotic taxa (FAPROTAX) (45).

Detailed descriptions of the WFPS calculations, soil physicochemical properties determination methods, crop enzyme activity measurements, extraction of epiphytic and endophytic DNA, and DI and DSI definitions are provided in Methods S1.

## RESULTS

A total of 288 samples were collected from six crop niches to characterize OTUs of bacteria. The OTUs were identified based on DNA sequences from the bacterial internal transcribed spacer region (V5-V7), which were amplified using specific primers with dual-barcoding. The amplified sequences were subsequently sequenced using Illumina Miseq, resulting in a total of 15,714,813 reads (Table S2). The average number of reads per sample was determined to be 54,565 (Table S3). Additionally, we have generated rarefaction curves, and high sequencing coverage was achieved in all samples, as shown by the rarefaction curves (Fig. S2). To account for variations in sequencing depth, we applied rarefaction to the data sets, and the specific rarefaction numbers utilized were set at 34,416.

### Characteristics of the bacterial community and factors influencing its structure

**Fig 1:**
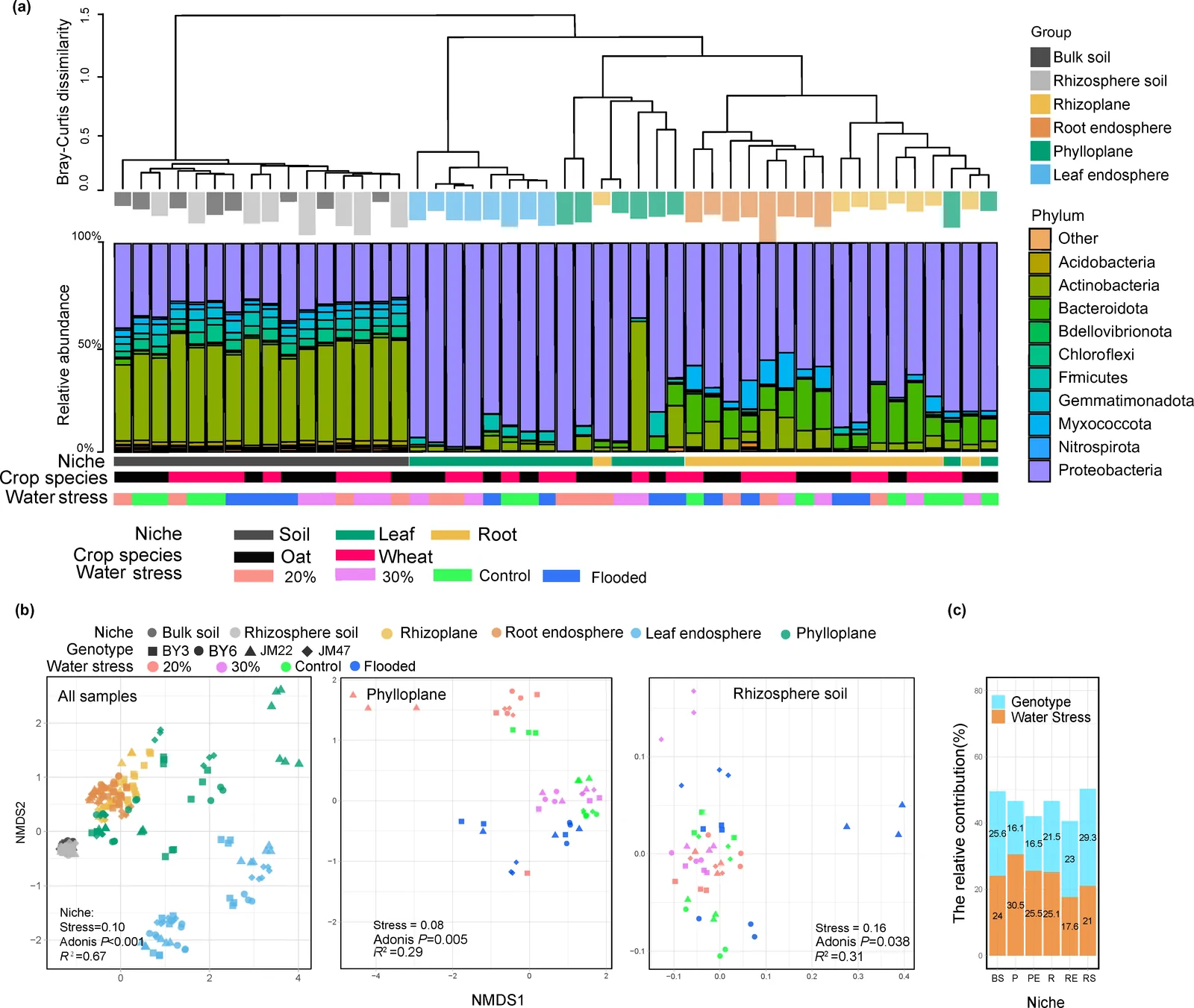
Host type takes precedence over water stress and genotype in shaping the crop microbiome. (**a**) Structure and taxonomic composition of bacterial communities varied across niches. Bray–Curtis distances were used to create a hierarchical clustering of bacterial OTUs from 48 samples. (To present the results clearly, 48 samples were randomly selected from the total of 288 samples. This means that eight samples were randomly selected from each crop compartment.) The Ward.D2 technique was used to cluster the samples. Low-abundance phyla with less than 1% of total sequences across samples were classified as "other." (**b**) Nonmetric multidimensional scaling (NMDS) ordinations of bacterial communities based on Bray–Curtis distances for all niche samples (*n* = 288), samples in phylloplane (*n* = 48), and rhizosphere soil (*n* = 48). (**c**) Relative contribution of species and water stress in different hosts. BS, bulk soil; P, phylloplane; LE, leaf endosphere; R, rhizoplane; RE, root endosphere; RS, rhizosphere soil.

Bacterial abundance varied significantly across host niches, with the highest abundance of Proteobacteria and Actinobacteria in bulk soils and rhizosphere soils, and in the four crop niches (phylloplane, leaf endosphere, rhizoplane, and rhizosphere soil), Proteobacteria, Actinobacteria, and Bacteroidota were the three most abundant bacteria (Fig. 1a; Fig. S3a and b). The number of core OTUs in the host niches (bulk soil excluded) was 460, and the largest numbers of specific OTUs were found in the rhizosphere soil (782) and leaf endosphere (641) (Fig. S3c). Additionally, a hierarchical clustering analysis indicated distinct clumping between leaf, root, and soil samples (Fig. 1a). The relative abundance of bacterial communities was analyzed, and it was found that bulk soil (41.1%) and rhizosphere soil (44.5%) possessed more Actinobacteria, whereas Proteobacteria were more abundant in the four crop niches (average content 65%–90%) (Fig. S4). Significant differences in composition were found between crop-associated and soil-associated bacterial communities.

The variation in the bacterial community was primarily described by compartment niche (*R* ^2^ = 66.7%, *P*＜0.001) and the interactions between host niche and water stress (*R* ^2^ = 6.9%, *P* = 0.029), based on the NMDS ordinations and nested PERMANOVA analysis of the entire data. Water stress (*R* ^2^ = 1.6%, *P* = 0.064) and species (*R* ^2^ = 1.3%, *P* = 0.172) did not have significant effects on the bacterial community structure (Fig. 1b; Table S4). There were significant differences in the relative contributions of water stress and crop genotype to the structure of bacterial communities in the different niches. Further investigation revealed distinct bacterial communities in the rhizosphere soil developed in each crop species (*R* ^2^ = 29.3%, *P* = 0.005), whereas distinct bacterial communities on the phylloplane arose in response to water stress (*R* ^2^ = 30.5%, *P* = 0.038). However, crop species and water stress had no significant effect on the bacterial community structure in the other crop niches (Fig. 1b; Table 1).

**TABLE 1:** Effect of crop species and water stress on bacterial community structure in different compartment niches based on PERMANOVA

Niche	Genotype		Water stress		Explain variation (%)
	*R* ^2^ (%)	Pr(>*F*)	*R* ^2^ (%)	Pr(>*F*)	
Bulk soil	25.6	0.102	24.0	0.138	49.6
Rhizosphere soil	29.3	0.005**^a^	21.0	0.126	50.3
Rhizoplane	21.5	0.201	25.1	0.082	46.6
Phylloplane	16.1	0.600	30.5	0.038*^a^	46.6
Root endosphere	23.0	0.251	17.6	0.663	40.6
Leaf endosphere	16.5	0.620	25.5	0.204	42.0

^*a*^ The significance is as follows: *P < 0.05, **P < 0.01.

### Host selection pressure reduces bacterial diversity and network complexity

**Fig 2:**
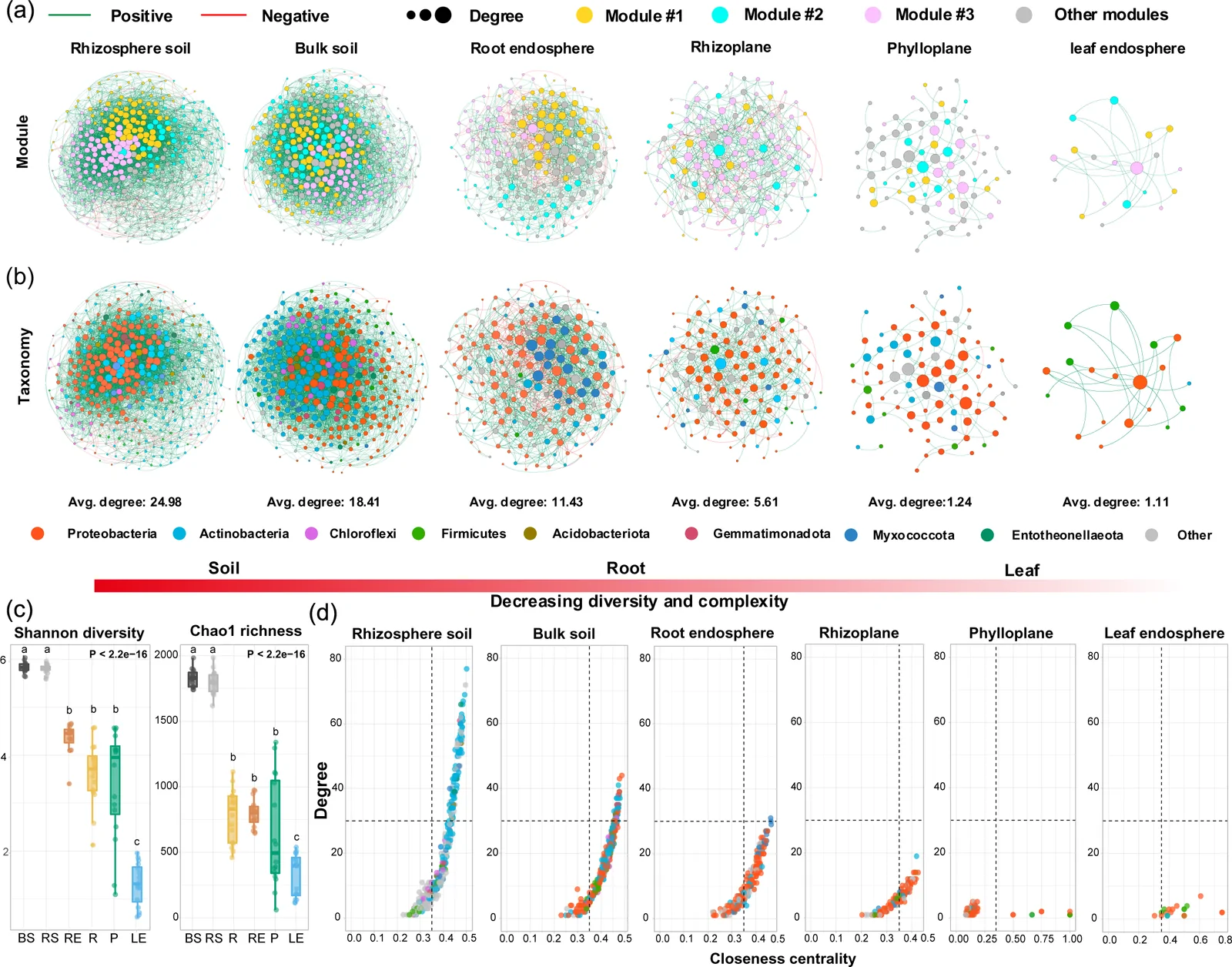
Reduced network complexity and bacterial diversity determined by crop hosts (**a and b**) Co-occurrence networks of the bacteria from soil to crop continuum in all samples (*n* = 288). (**c**) The alpha diversity of bacteria in different niches. BS, bulk soil; RS, rhizosphere soil; RE, root endosphere; P, phylloplane; R, rhizoplane; LE, leaf endosphere. (**d**) The “hub nodes” of the bacterial network distribution patterns in different niches.

The effect of host selection on crop microbial community diversity and network complexity was investigated. We observed that the alpha diversity and co-occurrence network complexity of bacterial communities (a network with a lower average degree is more simple) decreased progressively along the soil–root–leaf continuum (Fig. 2a and c). The network complexity and richness of bacterial communities progressively decreased from soils (the average degree in rhizosphere soil was 24.98 and 18.41 in bulk soil) to roots (11.43 in the root endosphere and 5.61 in the rhizoplane) and then to leaves (1.24 in the phylloplane and 1.11 in the leaf endosphere) (Fig. 2a and b). Moreover, Fig. 2c reveals that there was a marked fall in alpha diversity in the bacteria from soil to root and then to leaf (*P* < 2.2e-16). Further analysis revealed that the number of “hub nodes” (degree values > 30 and closeness centrality > 0.35) along the soil–root–leaf pathway decreased dramatically (Fig. 2d; Table S5). Differences in the taxonomic composition between the crop and soil compartments were also observed. There were more nodes in the soil niche belonging to Chloroflexi and more nodes in the crop niche belonging to Firmicutes (Fig. 2b and d). The phylloplane and the leaf endosphere had higher modularity. In addition, the phylloplane had a higher average path distance (Table S5).

The host niche (Shannon diversity, *F*_5, 237_ = 723.17, *P* < 2.2e-16) and water stress (*F*_5, 237_ = 11.98, *P* < 0.0001) had the largest impact on bacterial alpha-diversity, according to the linear mixed model analysis (Table S6). Additionally, the bacterial diversity in the other five niches was also significantly affected by water stress, except in the bulk soil (*F*_3, 32_= 1.0, *P* = 0.42). The rhizoplane (*F*_3, 32_= 104.5, *P* < 2.2e-16) and phylloplane (*F*_3, 32_= 773.1, *P* < 2.2e-16) diversities were significantly influenced by crop species (Table S7). Furthermore, in the compartment niches, the host exerted a considerable selection influence on the distribution patterns of the core taxa (OTUs were found in more than 80% of samples in different niches) and the dominant phylum (relative abundance＞1%). The number of core taxa was the highest in soils (bulk soil, 965; rhizosphere, 956) and then progressively lower in the roots (root endosphere, 402; rhizoplane, 304) and the leaves (phylloplane, 87; leaf endosphere, 54) (Table S8), and the lowest values were found in the leaf endosphere.

### Crop microbiome selection processes and potential sources

**Fig 3:**
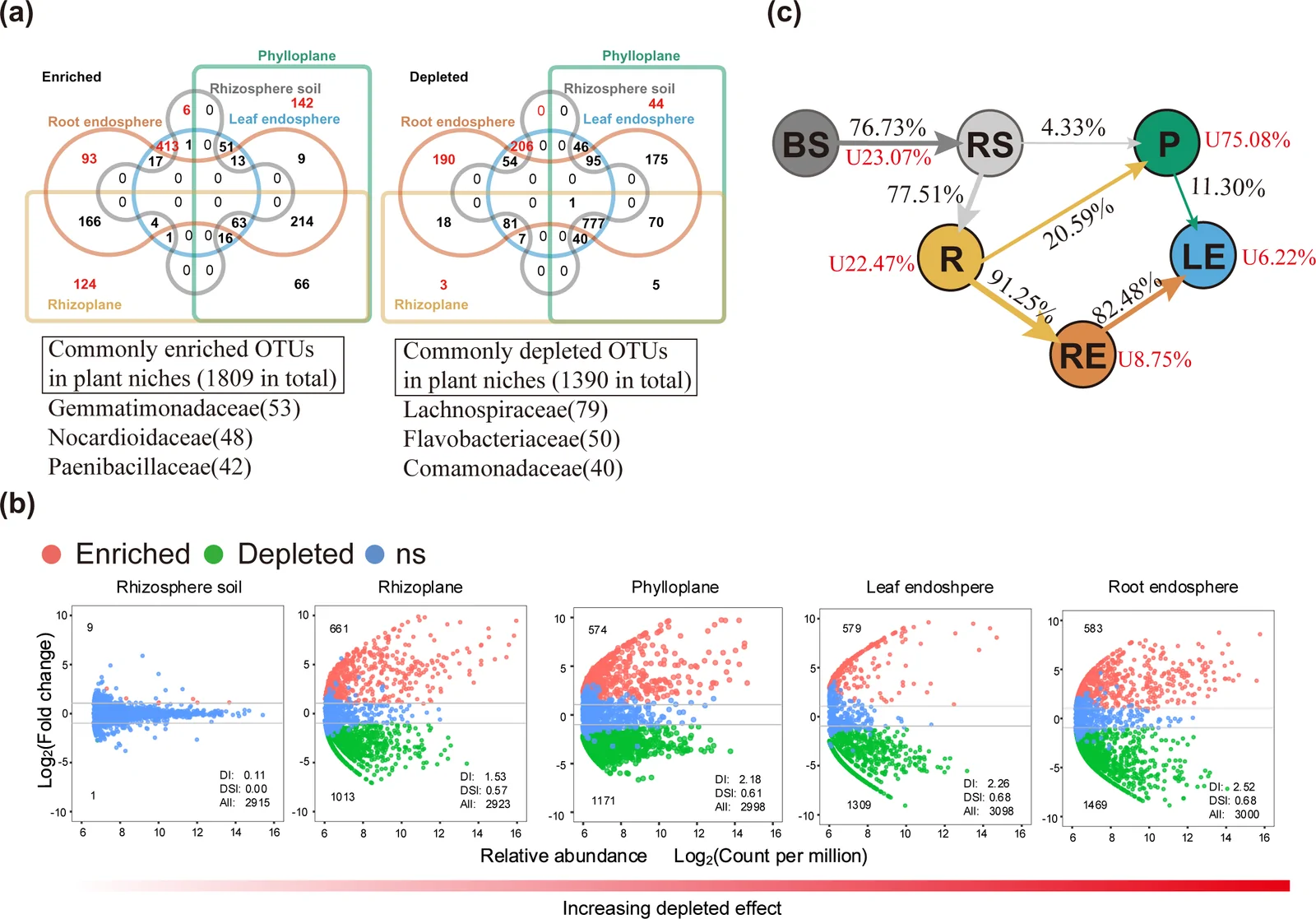
Through different compartment niches, bacterial communities associated with crops are gradually filtered and enriched, and they are mainly obtained from bulk soil. (**a**) The number of specific and shared enriched and depleted OTUs in each niche was shown with Venn diagrams, and the top three shared differential OTU taxonomies were shown. (**b**) Volcano plots were used to illustrate the enrichment and depletion patterns of the crop-associated bacterial microbiome for each compartment compared with the bulk soil. Each point represented an operational taxonomic unit (OTU) with a relative abundance greater than 0.1%, OTUs with red points were significantly enriched, blue points were not significantly enriched, and green points were depleted. OTU abundance was plotted on the *x*-axis [counts per million (CPM)], and fold change in abundance, when compared with bulk soil, is plotted on the *y* axis. (**c**) The source model of the crop microbiome constructed by FEAST showed the potential sources of crop-related bacterial communities. The thickness of the line represents the contribution of the source. “U” represents the percentage of the microbial community from unknown sources.

According to the differential abundance analysis, 31.2% of OTUs (1,390 out of 4,459 OTUs) were considerably depleted in the epiphytes and endophytes of the roots and leaves. These were primarily the Lachnospiraceae (79 OTUs), Flavobacteriaceae (50 OTUs), and Comamonadaceae (40 OTUs) families. In contrast, 40.6% of OTUs (1,809 out of 4,459 OTUs) were substantially enriched in the epiphytes and endophytes of the roots and leaves, with Gemmatimonadaceae (53 OTUs), Nocardioidaceae (48 OTUs), and Paenibacillaceae (42 OTUs) accounting for the majority (Fig. 3a and b). Furthermore, the leaf endosphere had the most specifically enriched OTUs (413), whereas the leaf endosphere and root endosphere had the most depleted OTUs (206 and 190, respectively) (Fig. 3a and b). The filtering and selection processes of microbial communities from rhizosphere soil to other niches, which were assessed with DI and DSI, were observed to increase progressively from the rhizosphere soil (0.11) to epiphytes (1.53–2.18) and endophytes (2.26–2.52) (Fig. 3b), indicating that the filtering and selection pressure of niches on microbial communities increased progressively along with the pathway from rhizosphere soil to epiphytes to endophytes. Based on the LDA effect size (LEfSe), the most important biomarker taxa were Gammaproteobacteria in leaves, Comamonadaceae in roots, and Alphaproteobacteria in soils (Fig. S5).

We performed a FEAST to pinpoint potential sources of bacterial communities detected in each host niche. In the rhizosphere soil, 76.93% of known sources came directly from the bulk soil, and 77.51% of known sources found on the rhizoplane were from the rhizosphere. In the root endosphere, 91.25% of known sources were from the rhizosphere, and in the leaf endosphere, 82.48% of known sources were from the root endosphere (Fig. 3c). Using FEAST, we found that bulk soil was the primary source of crop-related bacterial communities. The main sources of crop microbial communities were their neighboring compartment niches, which became progressively more filtered. In contrast, up to 75.08% of bacterial communities on the phylloplane had unknown sources, indicating that the phylloplane microbiome may be influenced by other factors.

### The microbial community significantly correlated with crop enzyme activity

**Fig 4:**
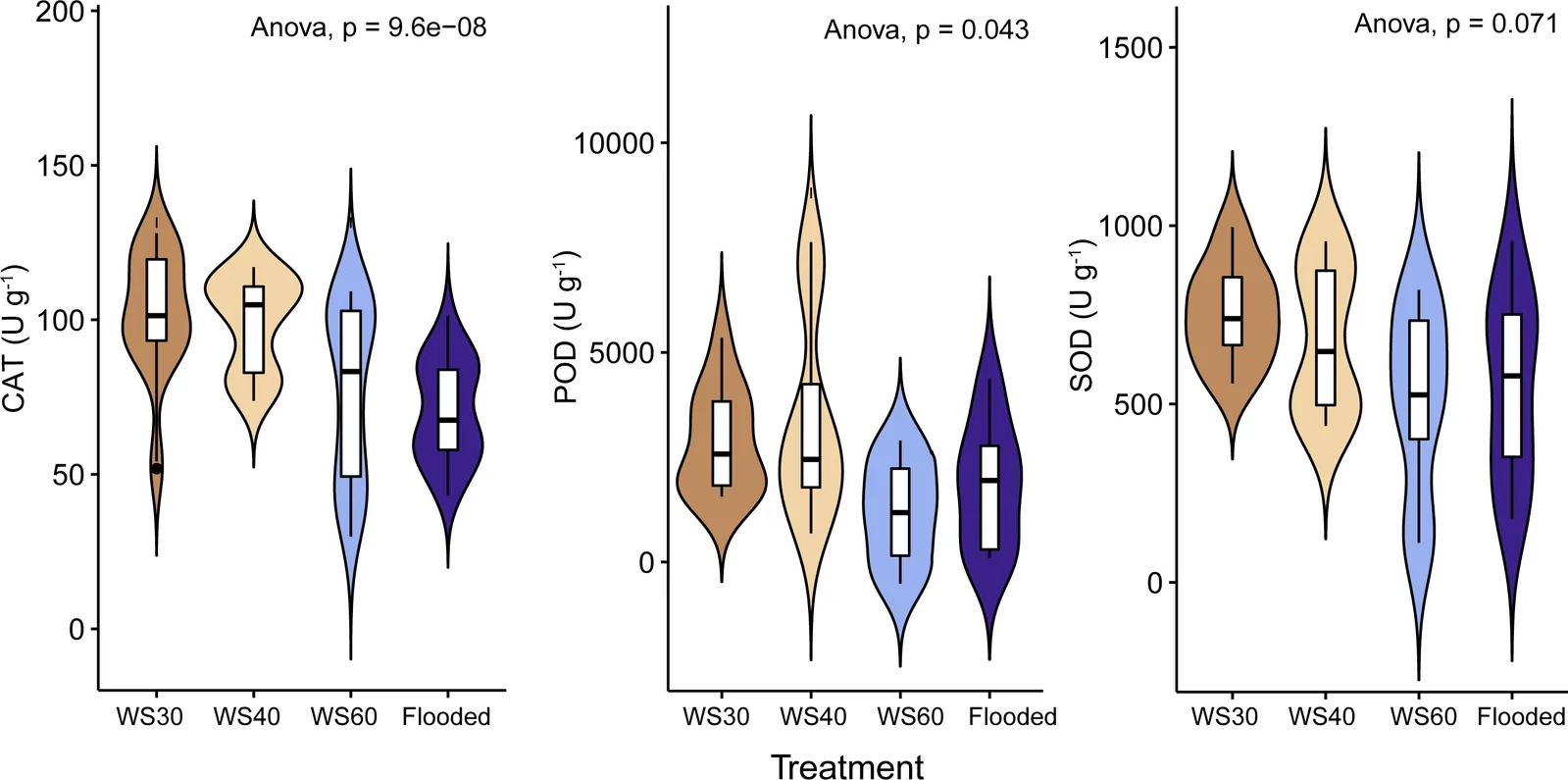
The activity of the catalase (CAT), peroxidase (POD), and superoxide dismutase (SOD) enzyme in crop leaves (*n* = 48). WS30, WS40, and WS60 represented samples taken where the soil moisture was 30, 40, or 60% of the soil’s water-filled pore space (WFPS), respectively.

In leaves, different water stress treatments resulted in a significant increase in CAT activity (*P* = 9.6e-08) and POD activity (*P* = 0.043) except for the flooded treatment condition (Fig. 4). Water stress increased the activity of leaf SOD compared with that of the control group, although not to a significant degree (*P* = 0.071). With the increase in stress intensity, the activity of these three enzymes also increases. Crop enzyme activity rises in response to water stress.

Through correlation analysis, it was found that the number of OTU positively correlated with enzyme activity was 26 in the root endosphere, only one in the phylloplane, and five in the leaf endosphere. The microbiota with the highest relative abundance that were significantly positively correlated with enzyme activity in the rhizoplane and the root endosphere were *Sorangium* (0.69%) and *Ensifer* (0.37%) in the leaf endosphere, *Asticcacaulis* (0.09%) in the rhizosphere soil, and *Blastococcus* (0.93%) in the bulk soil. The microbiota in all niches belonged to the Actinobacteria, Proteobacteria, Gemmatimonadota, and Myxococcota. Actinobacteria were detected in all crop niches except in the leaf endosphere (Table 2).

**TABLE 2:** Bacterial community significantly positively correlated with water stress^*a*^

Niche	Counts	Corresponding genus/family (count top 10)	Corresponding phylum (count top 3)
Phylloplane	1	*Micromonospora* (0.03%)	Actinobacteriota (1)
Leaf endosphere	5	*Ensifer* (0.37%), *Acidibacter* (0.35%), *Ralstonia* (0.28%), and *Methylobacterium* (0.07%)	Proteobacteria (5)
Rhizoplane	7	*Sorangium* (0.69%), Ilumatobacteraceae (0.02%), *Cellulosimicrobium* (0.01%), and *Gemmatimonas* (0.01%)	Myxococcota (1), Actinobacteriota (3), and Gemmatimonadota (1)
Root endosphere	26	*Sorangium* (0.69%)*, Ralstonia* (0.28%), *Blastococcus* (0.21%), *Solirubrobacter* (0.18%), Nitrosomonadaceae (0.17%), *Phyllobacterium* (0.02%), *Gaiella* (0.02%), Pseudohongiellaceae (0.02%), *Bauldia* (0.01%), and *Rubrobacter* (0.01%)	Myxococcota (3), Proteobacteria (11), and Actinobacteriota (6)
Rhizosphere soil	32	*Asticcacaulis* (0.09%), *Actinophytocola* (0.03%), *Porphyrobacter* (0.03%), Solirubrobacteraceae (0.03%), *Mesorhizobium* (0.02%), *Bryobacter* (0.01%), and Streptosporangiaceae (0.01%)	Proteobacteria (11), Actinobacteriota (9), and Myxococcota (4)
Bulk soil	65	*Blastococcus* (0.93%), *Solirubrobacter* (0.58%), Gemmatimonadaceae (0.48%), *Geodermatophilus* (0.32%), *Nocardioides* (0.24%), Geminicoccaceae (0.21%), and Rubrobacteriaceae (0.12%)	Actinobacteriota (34), Gemmatimonadota (3), and Myxococcota (3)

^*a*^ Bacterial communities with a relative abundance of less than 0.01% were not shown.

### Potential functions of bacterial microbiome in rhizosphere and soil phylloplane

Because in rhizosphere soil and phylloplane, microbial community assembly was significantly influenced by crop species and water stress, respectively, we predicted microbial functions in these two niches. Considering the indirect effect of water stress on root metabolites, only samples that experienced drought stress were subjected to functional analysis. Bacterial function putative investigations revealed that the top five abundant groups include chemoheterotrophy, aerobic chemoheterotrophy, aromatic compound degradation, ureolysis, and ligninolysis, all of which accounted for more than 66% of the respective total groups (Fig. S6).

## DISCUSSION

Exploring the mechanisms of the plant–microbe response to environmental changes is a critical task. This study is aimed to clarify the effects of water stress and crop species on the crop bacterial communities. We found that the main driver of crop microbiome formation was the host niche of the crop and that bacterial diversity and network complexity are determined by the host. The selection pressure on bacterial communities increased sequentially from soil to epiphytes to endophytes. The bacterial community was affected relatively little by water stress and crop species. Furthermore, we identified the likely source of the crop microbiome and the selection processes that influence it. We also performed a correlation analysis to search for microbial taxa that are associated with water stress. This study provides comprehensive empirical evidence concerning the response mechanisms of crop microorganisms under water stress and provides the necessary data to support the manipulation of crop microbiomes.

### Assembly processes of crop-associated bacterial communities under water stress

Our findings suggest that crop microbial assemblies are primarily determined by compartment niche rather than water stress or crop species (Table S4). Different gradients of water stress and crop species were not determinants of microbial assembly, implying that the various compartments of the crops contain specific bacteria less affected by water stress and crop species. Our conclusions are supported by the results of a study by Xiong et al. on the influence of different fertilization practices on bacterial communities at two separate sites yielded (13). Previous studies on the effects of drought on the composition of rice bacterial communities have found that the rice niche has a strong influence on bacterial composition, with each different bacterial species responding differently to drought stress in different rice niches (20). This finding also supports our conclusions. Cregger et al. found that the microbiomes largely differed across larger plant habitat types (46). Heil et al.’s study also concluded that the identity of the host species has a consistent effect on bacterial community structures (47). We conclude that different niches in the crop exert specific selection pressures, resulting in colonization by different groups of microbes.

The rhizosphere and phylloplane are the outermost layers of the plant environment, and they are also more sensitive to changes in biotic (crop species) and abiotic (water stress) factors. We found that crop species explained changes in the bacterial community in the rhizosphere soil, whereas water stress explained changes in the bacterial community on the phylloplane (Table 1). Our conclusions are supported by the results of studies on the rhizobial community of Amazonian dark soil plants and the phylogeny of annual plants, where plant species were identified as the main factor influencing the rhizobial community (48, 49). Rhizosphere bacterial communities subjected to the “rhizosphere effect” (50) developed significant differences at the species level, which were likely caused by differences in root metabolites across plant species (51, 52). Concerning the changes in the phylloplane bacterial communities, it is probable that differences in leaf surface water content caused by different levels of water stress, and hence variation in leaf surface pH, result in dramatically diverse bacterial communities (53).

This work also investigated bacterial abundance and network complexity in different crop niches. There was a constant decrease in these variables along the path from soil to epiphyte to endophyte. The lowest DI and DSI index values were identified in the root endosphere, whereas the leaf endosphere had the lowest microbial community abundance. This indicates that the host species have a significant selective influence on crop endophytes, which can be attributed to the immune system and metabolic secretions in the endophytes allowing the targeted selection of endophytic microorganisms and the retention of bacteria from a few taxa (54). In addition, among the six niches of interest, only the bacterial communities in the rhizosphere and phylloplane were significantly affected by crop species and water stress, and it is in these niches that the host–environment interaction occurs (55 – 57). Under water stress conditions, however, the leaf endosphere bacterial community changed drastically with the highest number of enriched OTUs and depleted OTUs (Fig. 3a). Agoussar et al.’s study also concluded that the primary location for isolating osmotic stress-resistant bacteria from plant tissues under drought conditions should be the leaf endosphere (58). The reaction of the leaf endosphere bacterial community to water stress suggests that future studies on crop water stress should pay more attention to this crop niche.

### Potential sources of bacterial communities in different crop compartment niches

Microbes are a link between the crop, soil, and environmental interactions, and learning about the potential sources and enrichment processes of microbes in different compartment niches of the plant is key to understanding plant adaptations to the ecological environment (59 – 61). Compared with the study of microbiome interactions between the soil and plant, little is known about how the microbiomes in different compartments of the plant interact. Source tracking analysis revealed that the main source of crop-associated bacteria communities was the soil, and these were filtered through the soil–root–leaf pathway. Unsurprisingly, 75% of bacteria from unknown sources were found in the phylloplane. As concluded in other studies (13, 62), it is likely that only low quantities of the microbiome were transported from the soil to the phyllosphere. In addition, we found that some members of the Gemmatimonadaceae (Gemmatimonadota), Nocardioidaceae (Actinobacteria), and Paenibacillaceae (Firmicutes) families were remarkably rich in crop niches, but other families, including Lachnospiraceae (Firmicutes), Flavobacteriaceae (Bacteroidota), and Comamonadaceae (Proteobacteria), were greatly depleted. This is consistent with most other studies where, in general, the abundance of Gram-positive phyla such as Actinobacteria and Firmicutes increases in water-limited soils, whereas the relative abundance of Proteobacteria and Bacteroidetes of the Gram-negative phyla decreases (63, 64). Adding to the complexity, Firmicutes appear in both enriched and depleted phyla. This can probably be attributed to the diverse tolerance mechanisms and life strategies of bacteria inhabiting different ecological niches (64) or their distinctive morphologies (cell membrane structures, etc.). These results may also be influenced by the relative abundance of each sub-phyla in different studies (63). We discovered that the root endosphere, not the phylloplane, was the primary source of leaf endosphere bacteria, whereas the root endosphere bacteria mostly originate from the rhizoplane, from where they are transmitted via the internal crop tissues (xylem or nutrient-rich intracellular spaces) to the above-ground section of the crop (65). In addition, endophytes are under higher selection pressure from hosts than epiphytes. Hence, the sources of epiphytes are mostly unknown but may come from other pathways such as air, rainwater, or the seed (56, 66).

### Bacterial communities significantly associated with water stress

Microorganisms co-occurring with crops could influence the resistance of crops to water stress, but tolerance levels vary (58, 67). Higher antioxidant activity is usually correlated with increased plant stress resistance (68). We used the intensity of enzyme activity to characterize the level of drought stress and isolated crop symbiotic bacteria from stress conditions that were significantly and positively correlated with crop enzyme activity. Our results show that bacterial communities significantly associated with water stress belonged to four phyla: Actinobacteria, Proteobacteria, Gemmatimonadota, and Myxococcota. Actinobacteria was detected in five niches but not within the leaf endosphere (Table 2). In general, Actinobacteria, Proteobacteria, and Gemmatimonadota all include bacteria that are resistant to water stress (4, 69). In particular, Actinobacteria show a high adaptation to aridity because they can synthesize and accumulate ribosomes under drought stress, making them more efficient at obtaining nutrients after the drought (70). Only Proteobacteria were detected in the leaf endosphere. Incidentally, bacteria growing at high osmotic pressure were more frequently isolated in the leaf endosphere than in the rhizosphere or the rhizoplane (58). In contrast, Myxococcota appeared only in association with crop roots (the root endosphere, rhizoplane, and rhizosphere soil). Based on these findings, we suggest that resistance to water stress is a quality inherent to different parts of the crop, which is further evidence that the host is the most significant factor in bacterial selection. Identifying bacterial communities strongly linked to crop enzyme activity can provide information to aid in the isolation of bacteria for osmotic stress resistance.

### Bacterial community functions in rhizosphere soil and phylloplane

Predictions of bacterial community function were made for bacteria in the phylloplane and rhizospheric soils by FAPROTAX, as water stress significantly affected bacterial community assemblages in both niches. We only examined samples under drought stress (WS30, WS40) to get an overview of bacterial community function under drought. The ecological functions of the core bacterial community predicted by FAPROTAX involve enhancing the ability of crops to absorb nutrients and promoting nutrient cycling in crop systems, which affect the physiological and biochemical activities of crops, help crop growth, maintain soil ecological balance, and improve crop drought tolerance.

### Conclusions

In conclusion, our study reveals the relative contributions of different crop compartment niches, genotypes, and water stress to the bacterial assemblies in wheat and oats. Our findings show that the microbial assemblage was largely determined by compartment niche rather than crop genotype or water stress. Along the path from soil to epiphyte to endophyte, bacterial diversity was subject to sequentially increasing selection pressure from the host, resulting in progressive decreases in network complexity. In addition, microbial source tracking analysis showed that crop endophytes select most taxa from nearby species pools and that the root endophytes are the main potential source of leaf endophytes. We also observed *Gemmatimonadaceae, Nocardioidaceae,* and *Paenibacillaceae* microbial species selectively colonizing the crop. We also detected bacterial communities significantly associated with crop enzyme activity, suggesting that resistance to water stress is inherent to different crop compartments. We also predicted the ecological functions of rhizosphere soils and phylloplane core microbial communities. This finding could assist with the isolation of high-osmotic-stress bacterial communities. Our findings contribute to a better understanding of crop microbiome assembly patterns under water stress and provide crucial information concerning the use of crop microbiomes by crops to resist water stress.

## Data Availability

Raw sequence data have been deposited in the public database of the National Center for Biotechnology Information (NCBI) under BioProject accession number PRJNA855045. The R scripts used in data analyses are available from the corresponding author upon request.

## SUPPLEMENTAL MATERIAL

Fig. S1 to S6, Tables S1 to S8 spectrum.00068-23-s0001.docx Detailed description of experiment trial, experimental methods, statistical analysis, figures, and tables.

ASM does not own the copyrights to Supplemental Material that may be linked to, or accessed through, an article. The authors have granted ASM a non-exclusive, world-wide license to publish the Supplemental Material files. Please contact the corresponding author directly for reuse.
