# Supplementary material for: Drought-resistant trait of different crop genotypes determines assembly patterns of soil and phyllosphere microbial communities
Source: Microbiol Spectr. 2023 Sep 27;11(5):e00068-23. doi: 10.1128/spectrum.00068-23 (PMC10581042; doi:10.1128/spectrum.00068-23)
Supplement: Fig. S1 to S6, Tables S1 to S8 — Detailed description of experiment trial, experimental methods, statistical analysis, figures, and tables. [file spectrum.00068-23-s0001.docx]

***Microbiology Spectrum***

**Supplementary materials**

**Drought-resistant trait of different crop genotype determines assembly patterns of soil and phyllosphere microbial communities**

Baobei Guo ^a,b,^ , Hong Zhang ^a*^ , Yong Liu ^a^ , Jianwen Chen ^a^ ,Junjian Li ^a*^

*^a^ Institute of Loess Plateau, Shanxi University, Taiyuan, Shanxi, 030006, China*

*^b^ Pomology Institute, Shanxi Agricultural University, Taiyuan, Shanxi, 030006, China*

*Corresponding authors: zhanghong@sxu.edu.cn; lijunjian@sxu.edu.cn

**Supplementary methods**

Detailed description of experiment trial, experimental methods, and statistical analysis.

Water-filled pore space (WFPS,%) was calculated according to the following relationship, $WFPS=(SWC\times BD)/(1-\left( BD/PD \right))$, where SWC is for soil water content (g g-1), BD stands for bulk density (g cm-3), and PD stands for particle density (assume to be 2.65 g cm-3) (Franzluebbers 1999). When carried out water stress, after adjusting the WFPS of the soil, the soil moisture content was measured with ML3 Thetakit (Delta-T Devices, UK) to adjust the soil moisture in time by referring to the values displayed.

The following physicochemical properties of the three types of soil were measured: (i) the available K (AK) in the soil was extracted with ammonium acetate and determined using flame photometry; (ii) the available P (AP) in the soil was extracted with sodium bicarbonate and then determined using the molybdenum blue method; (iii) the soil moisture was determined using oven drying method; (iv) the ammonium nitrogen (NH+ 4 -N) was determined by the Hissink method; (v) the nitrate nitrogen (NO- 3-N) was extracted with calcium sulfate and determined by double wavelength UV spectrophotometry method; (vi)the soil organic matter (OM) was determined using the potassium dichromate volumetric method; (vii) the soil pH was determined using a FiveEasy FE20 pH detector (METTLER TOLEDO, Switzerland) at a soil-to-water ratio of 1:5; (viii) the total N (TN) was determined via Kjeldahl digestion.

Catalase (CAT) Activity Assay Kit, Micromethod, Peroxidase (POD) Activity Assay Kit, Micromethod, and Superoxide Dismutase (SOD) Activity Assay Kit, Micromethod (Sangon Biotech, Shanghai) was used to measure (SOD) peroxidase (POD) and catalase (CAT) in the leaves according to manufacturer’s instructions. The multimode plate reader VICTOR® Nivo™ (PerkinElmer, GER) was used for detection.

Epiphytic DNA was collected as before (1) by taking 2g each of roots and leaves (rhizosphere soil carefully removed from the root firstly) and submerged in 0.1 M Phosphate Buffered Saline (pH 8.0). To collect microbial cells, they were sonicated at 40 kHz for 1 minute and agitated at 250 rpm on a shaker for 2 minutes. The buffer was then filtered using a 0.22 μm-pore filter after being repeated 3 times. According to the manufacturer's recommendations, these filters were used for DNA extraction using the PowerSoil DNA Isolation Kit (MO BIO Laboratories, Carlsbad, CA, USA). For endophytic DNA, the leaves or roots from which epiphytic microorganisms had been collected were first further washed with sterile H_2_O, then rinsed twice with 70% ethanol, then soaked in 70% ethanol for 30 seconds, and finally washed three times with sterile H_2_O (2). After grinding the treated leaf and root tissues with liquid nitrogen in sterile mortars and pestles, endophytic DNA was isolated from the 0.5 g powder using the E.Z.N.A.® soil DNA Kit (Omega Bio-tek, Norcross, GA, U.S.).

To assess the degree of impact of each enrichment process, we defined the “depleted index” (DI = Depleted OTUs / Enriched OTUs ) and the "dissimilarity index" [DSI = (Enriched OTUs + Depleted OTUs)/ The total OTUs (relative abundance > 0.1%)]. Thus, the higher the DI score the more OTUs were deleted, which indicates a greater depletion effect. the higher the DSI index, the greater the difference between this niche and bulk soil.

**References**

1. Khan MU, Li P, Amjad H, Khan AQ, Arafat Y, Waqas M, Li Z, Noman A, Islam W, Wu L, Zhang Z, Lin W. 2019. Exploring the Potential of Overexpressed OsCIPK2 Rice as a Nitrogen Utilization Efficient Crop and Analysis of Its Associated Rhizo-Compartmental Microbial Communities. Int J Mol Sci 20:E3636.

2. Samad A, Trognitz F, Compant S, Antonielli L, Sessitsch A. 2017. Shared and host-specific microbiome diversity and functioning of grapevine and accompanying weed plants. Environ Microbiol 19:1407–1424.

**Supplementary figures**


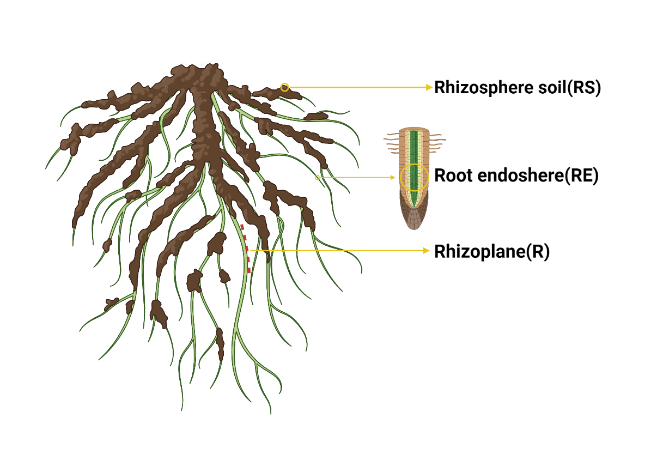

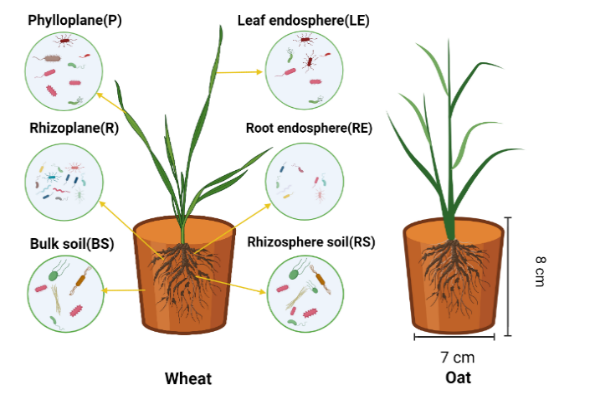

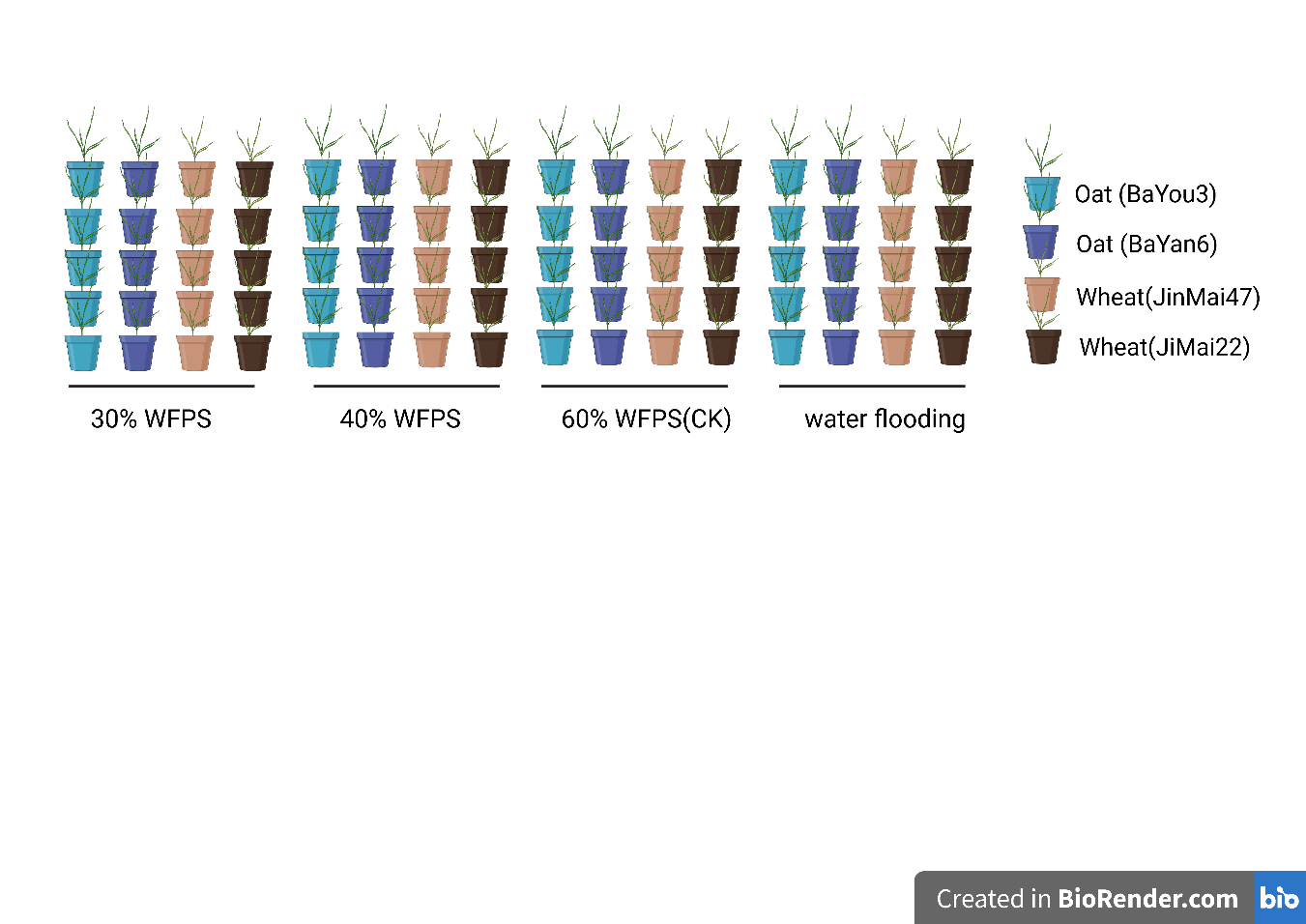


**Figure S1** Experimental design and the layout of samples in terms of water stress treatments, and plant compartment niches. Icons made by Biorender from https://biorender.com/.


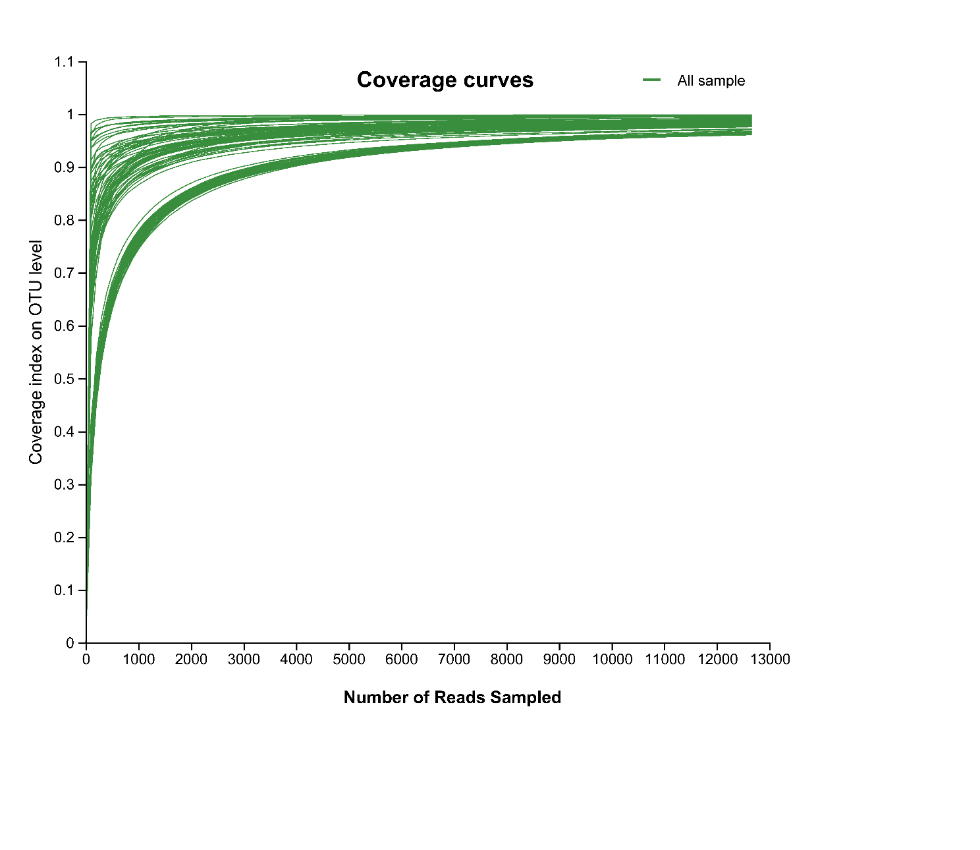


**Figure S2**. Rarefaction curves of all samples.


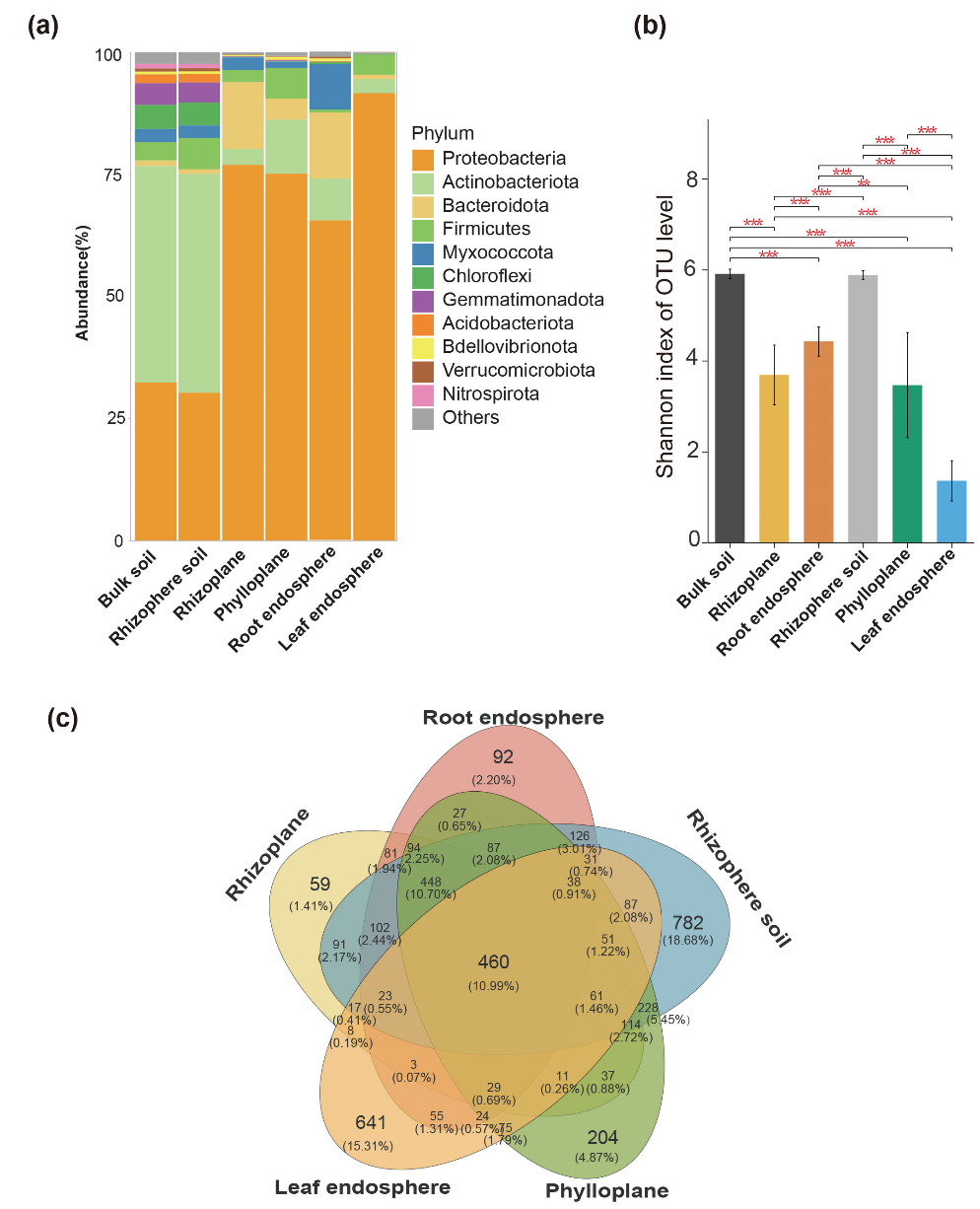


**Figure S3** Structure of bacterial communities in different niches. (a) Bacterial 16S rRNA gene reads from 6 niches accounted for an average of more than 1% of the relative abundance of phyla. (b) Bacterial alpha diversity in different niches (Grouped column plot with asterisk bracket to show significant differences. Asterisk denotes statistically significant differences ** *P* <0.01; *** *P* <0.001). (c) Venn diagrams show the shared and specific bacterial OTUs in different niches.


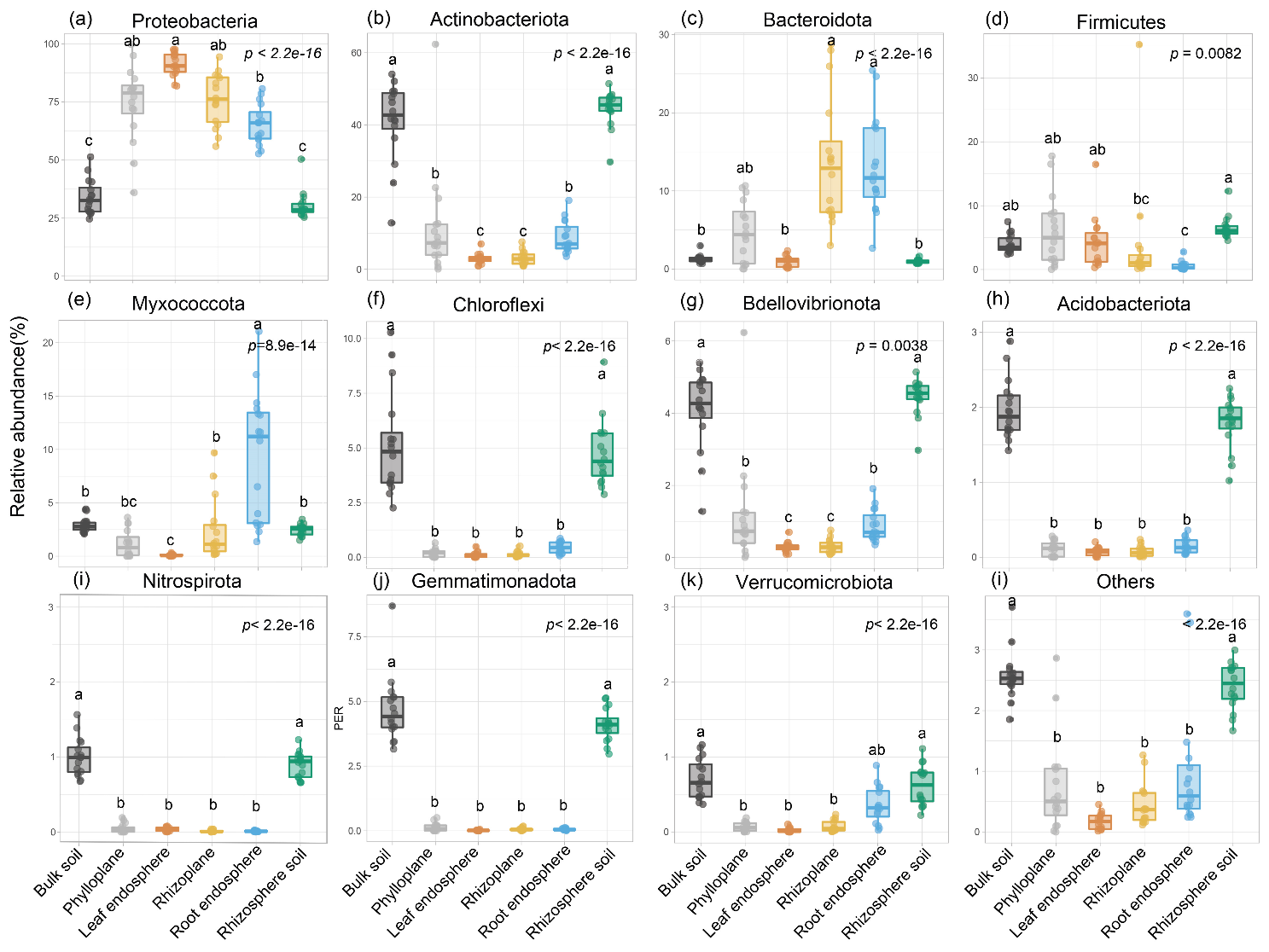


**Figure S4** In various environments, the relative abundance of dominating phyla/classes differed. (a-i) In distinct environments, the relative abundance of dominating phyla/classes. A substantial difference was indicated by different letters above the boxes.


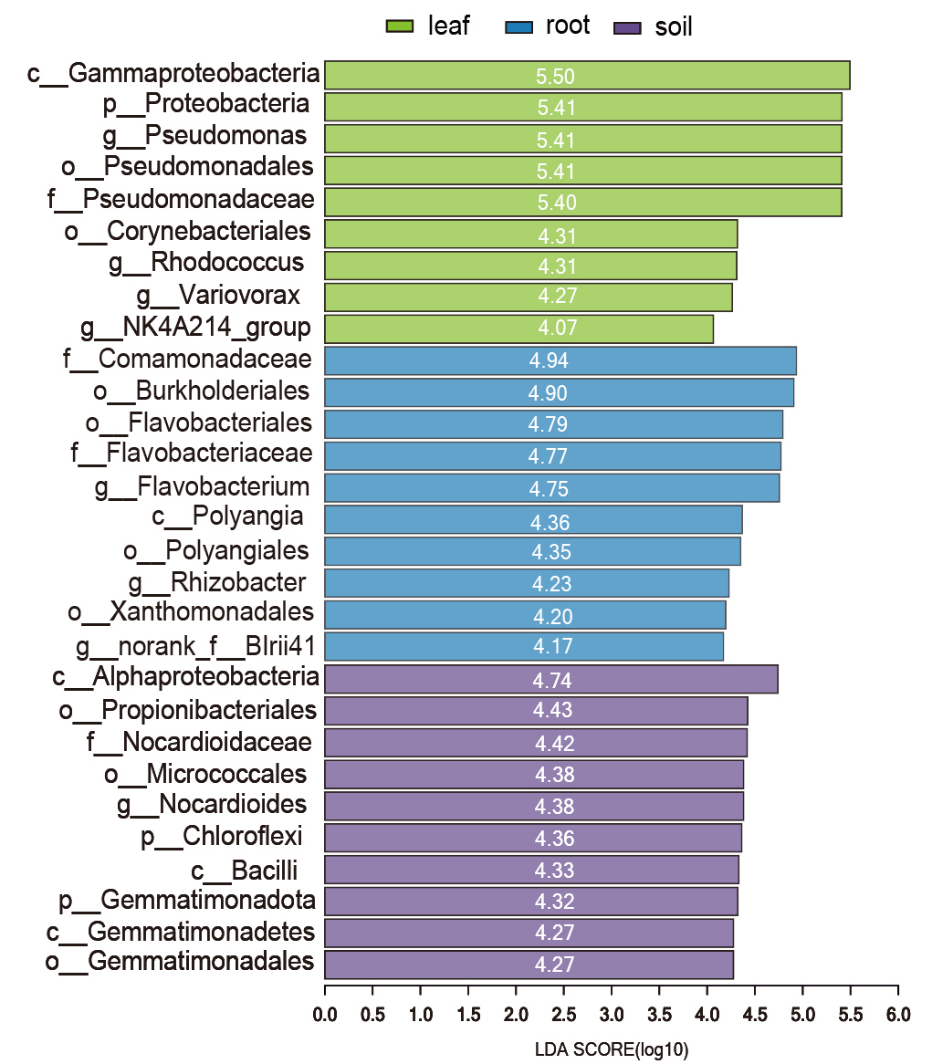


**Figure S5** LEfSe identified biomarker taxa associated with leaves, roots, and soil, respectively. Only the top 10 most specific biomarkers taxa were shown.


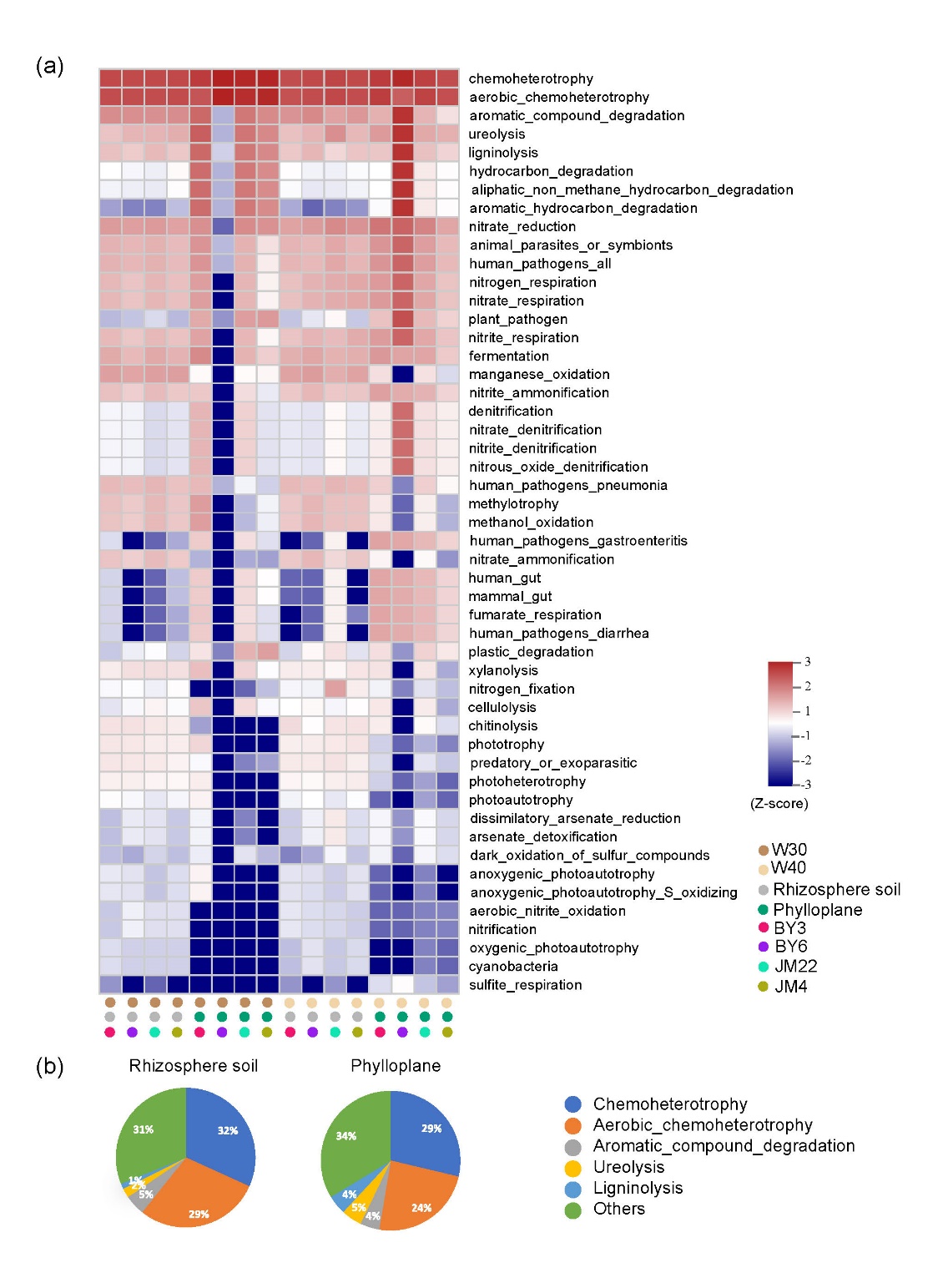


**Figure S6** The potential functional groups of bacterial communities among rhizosphere soil and phylloplane. (a) Heat map exhibiting the relative abundance of dominant function groups (top50) across rhizosphere soil (n=24) and phylloplane (n=24). WS30, WS40 represented samples taken where the soil moisture was 30, 40 of soil's water-filled pore space (WFPS), respectively. (b) Composition of dominant function on rhizosphere soil and phylloplane.

**Supplementary tables**

Table S1 The soil properties.

| Item | Value |
| --- | --- |
| AK | 254 mg kg^-1^ |
| AP | 17.3 mg kg^-1^ |
| Moisture | 9.23% |
| NH+ 4 -N | 3.195 mg kg^-1^ |
| NO- 3-N | 1.585 mg kg^-1^ |
| OM | 19.8 g kg^-1^ |
| pH | 8.3 |
| TN | 0.106% |

*Abbreviation: AK Available potassium, AP Available phosphorus, Moisture soil water content, NH+ 4 -N Ammonium nitrogen, NO- 3-N Nitrate nitrogen, OM organic matte, TN total content of nitrogen.*

Table S2 Optimising sequence information.

| Amplified Region | Total samples | Sequences | Bases(bp)b | Average Length |
| --- | --- | --- | --- | --- |
| 799F_1193R | 288 | 15714813 | 5918756415 | 376 |

a The number of optimized sequences (after double-ended sequence quality control splicing).

b The number of bases in the optimized sequence.

Table S3 Information statistics for each of the samples from the different crop niches.

| Niche | Sequence number | Base number | Mean length | Minimum sequence length | Maximum sequence length |
| --- | --- | --- | --- | --- | --- |
| Bluk soil | 54167±11616 | 20400516±4373617 | 376.622±0.324 | 202±1.31 | 475±29.83 |
| Phylloplane | 51118±15063 | 19261364±5673583 | 376.826±0.732 | 227±36.74 | 447±53.65 |
| Leaf endosphere | 49318±9967 | 18619766±3686650 | 377.547±0.117 | 291±56.87 | 445±41.66 |
| Rhizoplane | 56880±10610 | 21399468±4000831 | 376.188±0.534 | 205±14.02 | 444±46.22 |
| Root endosphere | 61503±12404 | 23128552±4651707 | 376.103±0.481 | 202±2.84 | 472±37.7 |
| Rhizosphere soil | 54406±8863 | 20497760±3342814 | 376.745±0.301 | 201±1.03 | 455±24.68 |
| Average | 54565 | 20551237 | 376.65 | 221 | 456 |

Table S4 Effects of host niche, crop species, and water stress on bacterial community based on PERMANOVA.

| Variables | *F* value | df | *R*^2^(%) | Pr( > *F*) |
| --- | --- | --- | --- | --- |
| Host niche | 40.96 | 5 | 66.7 | 0.0001*** |
| Genotype | 1.339 | 3 | 1.3 | 0.172 |
| Water stress | 1.650 | 3 | 1.6 | 0.064 |
| Host niche **×**Genotype | 1.099 | 15 | 5.4 | 0.291 |
| Host niche **×** Water stress | 1.412 | 15 | 6.9 | 0.029* |
| Water stress **×**Genotype | 1.176 | 9 | 3.4 | 0.224 |

*The significance of host niche, crop genotypes, and water stress on the bacterial community was tested with PERMANOVA or nested PERMANOVA (based on Bray-Curtis distances). The model explains a total of 85.3% of the variation in bacterial community structure for all samples.*

Table S5 Characteristics of the bacterial co-occurrence network in each compartment niche

| Niche | Node | Hub node^a^ | Positive edge | Negative edge | Average degree | Modularity^b^ | Average clustering coefficient^c^ | Average path distance^d^ |
| --- | --- | --- | --- | --- | --- | --- | --- | --- |
| Rhizosphere soil | 356 | 137 | 4383 | 88 | 24.98 | 0.250 | 0.215 | 2.574 |
| Bulk soil | 356 | 38 | 3325 | 16 | 18.41 | 0.213 | 0.087 | 2.464 |
| Root endosphere | 184 | 2 | 820 | 254 | 11.43 | 0.369 | 0.217 | 2.796 |
| Rhizoplane | 135 | 0 | 328 | 79 | 5.61 | 0.355 | 0.079 | 3.002 |
| Phylloplane | 88 | 0 | 81 | 3 | 1.11 | 0.769 | 0.014 | 7.874 |
| Leaf endosphere | 19 | 0 | 23 | 0 | 1.24 | 0.511 | 0.200 | 2.396 |

*^a^ A hub node is defined as a node that has a high degree (>30) and closeness centrality (>0.35) in the network.*

*^b^ Degree of nodes tending to distinguish between different network modules.*

*^c^ Degree of nodes tending to cluster together.*

*^d^ The length of the shortest path between two nodes within the network.*

Table S6 Effects of host niche, water stress, and crop species on bacterial alpha diversity based on linear mixed model(LMM).

| Variables | Shannon diversity | | Chao1 richness | |
| --- | --- | --- | --- | --- |
|  | *F* value | *Pr* (>*F*) | *F* value | *Pr* (>*F*) |
| Host niche | F5,237=723.17 | < 2.2e-16 | F5,237=361.88 | < 2.2e-16 |
| Water stress | F3,237=11.98 | 2.5e-07 | F3,237=18.45 | 8.6e-11 |
| Genotype | F3,2=3.12 | 0.25 | F3,2=3.20 | 0.25 |
| Host niche ×Water stress | F15,237=5.76 | 4.3e-10 | F15,237=6.77 | 4.0e-12 |
| Host niche × Genotype | F15,237=3.91 | 2.9e-06 | F15,237=1.99 | 0.02 |
| Water stress× Genotype | F9,237=6.70 | 1.5e-08 | F9,237=3.87 | 1.3e-4 |

*In the linear-mixed model, significance was determined using ANOVA with Satterthwaite approximation of the degrees of freedom. The model explains 93.1% and 87.6% of the marginal variance(only owing to fixed variables) in bacteria Shannon diversity and Chao1 richness, respectively.*

Table S7 Bacteria alpha diversity is explained by different factors within each niche

| Niche | Water stress | | Genotype | | | Water stress × Genotype | | | Explained marginal  Variation (%) |
| --- | --- | --- | --- | --- | --- | --- | --- | --- | --- |
|  | *F* value | *Pr* (>*F*) | | *F* value | *Pr* (>*F*) | | *F* value | *Pr* (>*F*) |  |
| Bulk soil | 1.0 | 0.4232 | | 6.8 | 0.0012 | | 11.5 | 8.7e-08 | 72.9 |
| Rhizosphere soil | 13.2 | 9.2e-06 | | 1.6 | 0.2042 | | 8.1 | 4.0e-06 | 71.1 |
| Rhizoplane | 371.4 | < 2.2e-16 | | 104.5 | < 2.2e-16 | | 119.6 | < 2.2e-16 | 98.1 |
| Root endosphere | 29.7 | 2.3e-09 | | 124.9 | 0.0080 | | 66.4 | < 2.2e-16 | 95.7 |
| Phylloplane | 1343.3 | < 2.2e-16 | | 773.1 | < 2.2e-16 | | 1407.4 | < 2.2e-16 | 99.8 |
| Leaf endosphere | 138.9 | < 2.2e-16 | | 28.6 | 0.034 | | 104.5 | < 2.2e-16 | 96.8 |

*Drivers of bacterial alpha diversity in each niche were quantified using the Shannon diversity. Significance was assessed using type II ANOVA with Satterthwaite approximation of the degrees of freedom in a linear-mixed model.*

Table S8 Core taxonomy of bacterial community in each niche

| Niche | Counts | Corresponding genus/family (Abundance Top 10 OTUs) | Corresponding Family  (Count Top 3) | Corresponding Phylum  (Count Top 3) | Most abundant  OTU |
| --- | --- | --- | --- | --- | --- |
| All niches | 109 | *Pseudomonas*(15.49%,3.47%,2.20%) Comamonadaceae(3.46%) *Variovorax*(1.70%) *Rhodococcus*(1.57%) *Flavobacterium*(1.48%) *Acidovorax*(1.44%) *Cellvibrio*(1.09%) *Escherichia-Shigella*(1.06%) | Comamonadaceae(12) Rhizobiaceae(12) Xanthomonadaceae(8) | Proteobacteria(74) Actinobacteriota(24)  Bacteroidota(4) | *Pseudomonas* (15.49%) |
| Phylloplane | 87 | *Pseudomonas*(12.89%,8.27%,5.99%,2.00%) *Rhodococcus*(7.52%) *Rahnella1*(6.11%) Comamonadaceae(2.56%) *Escherichia-Shigella*(2.51%) Enterobacteriaceae(2.53%) *Rhizobacter*(1.91%) | Comamonadaceae(10) Pseudomonadaceae (8) Rhizobiaceae (7) | Proteobacteria(60) Actinobacteriota(18) Firmicutes(5) | *Pseudomonas* (12.89%) |
| Leaf endosphere | 54 | *Pseudomonas*(74.03%,1.17%) *Variovorax*(7.72%) *Rhodococcus*(1.61%) *Escherichia-Shigella*(1.53%) *Lactococcus*(1.04%) *Raoultella*(0.85%) Enterobacteriaceae(0.62%) *Klebsiella*(0.58%) *Bifidobacterium*(0.55%) | Pseudomonadaceae(8) Enterobacteriaceae(7) Comamonadaceae(4) | Proteobacteria(36) Firmicutes(8) Actinobacteriota(7) | *Pseudomonas* (74.03%) |
| Rhizoplane | 304 | Comamonadaceae(10.15%) *Rahnella1*(9.10%) *Pseudomonas*(7.02%,4.94%,4.06%) *Flavobacterium*(4.64%) *Acidovorax*(3.86%) Enterobacteriaceae(3.24%) *Cellvibrio*(2.65%) *Flavobacterium*(2.18%) | Flavobacteriaceae(26) Comamonadaceae(24) Rhizobiaceae(24) | Proteobacteria(175) Actinobacteriota(60) Bacteroidota(30) | Comamonadaceae (10.15%) |
| Root endosphere | 402 | *Pseudomonas*(7.73%) Comamonadaceae(6.47%) *Rhizobacter*(3.55%) *Flavobacterium*(2.81%,2.36%) *Acidovorax*(2.73%) *Cellvibrio*(2.45%) *Sorangium*(2.17%) *Devosia*(2.19%) Xanthomonadaceae(2.08%) | Rhizobiaceae(27) Pseudomonadaceae(24) Comamonadaceae(22) | Proteobacteria(217) Actinobacteriota(67) Bacteroidota(36) Myxococcota(36) | *Pseudomonas* (7.73%) |
| Rhizosphere soil | 956 | *Blastococcus*(4.10%) *Solirubrobacter*(2.90%) 67-14(2.53%) c__MB-A2-108(1.80%) Gemmatimonadaceae(2.22%) *Bacillus*(1.78%) *Arthrobacter*(1.67%) *Geodermatophilus*(1.34%) *Sphingomonas*(1.23%) *Gaiella*(1.05%) | Nocardiopsaceae(41) Geodermatophilaceae(28) Bacillaceae(22)  Thermoanaerobaculaceae(22) | Proteobacteria(294) Actinobacteriota(267) Chloroflexi (75) | *Blastococcus* (4.10%) |
| Bulk soil | 965 | *Blastococcus*(4.59%) *Solirubrobacter*(2.41%) Gemmatimonadaceae(2.29%) *Arthrobacter*(2.02%) 67-14(1.99%) c__MB-A2-108(1.76%) *Geodermatophilus*(1.55%) *Sphingomonas*(1.06%) *Blastococcus*(1.05%) Geminicoccaceae(0.094%) | Nocardioidaceae(38) Geodermatophilaceae(32) Pseudonocardiaceae(24) | Actinobacteriota(319) Proteobacteria(309) Chloroflexi (67) | *Blastococcus* (4.59%) |

*The counts of OTUs were detected in more than 80% of samples in different niche*
